# Central Neuropathic Pain in a Patient with Multiple Sclerosis Treated Successfully with Topical Amitriptyline

**DOI:** 10.1155/2012/471835

**Published:** 2012-07-18

**Authors:** David J. Kopsky, Remko Liebregts, Jan M. Keppel Hesselink

**Affiliations:** ^1^Clinical Operations, Institute for Neuropathic Pain, Vespuccistraat 64-III, 1056 SN Amsterdam, The Netherlands; ^2^Department of Anesthesiology, Pain and Palliative Medicine, Radboud University Nijmegen Medical Centre, Postbus 9101, 6500 HP Nijmegen, The Netherlands; ^3^Research and Development, Institute for Neuropathic Pain, Spoorlaan 2a, 3735 MV Bosch en Duin, The Netherlands

## Abstract

Central neuropathic pain in patients with multiple sclerosis (MS) is a common debilitating symptom, which is mostly treated with tricyclic antidepressants or antiepileptics. Unfortunately, the use of these drugs is often limited due to adverse events. We investigated the analgesic effect of topical amitriptyline 5% and 10% cream in a patient with central neuropathic pain due to MS. The analgesic effect of topical amitriptyline cream on neuropathic pain was dose related. To evaluate whether this analgesic effect is due to the active compound or placebo, we conducted a double-blind placebo-controlled n-of-1 study with amitriptyline 5% cream and placebo. The instruction was to alternate the creams every week following the pattern ABAB, with an escape possibility of amitriptyline 10% cream. The result was a complete pain reduction after application of cream B, while most of the time cream A did not reduce the pain. The patient could correctly unblind both creams, determining B as active. She noted that in the week of using the active cream no allodynia was present, with a carryover effect of one day.

## 1. Introduction

Central neuropathic pain in patients with multiple sclerosis (MS) is a common debilitating symptom, which is mostly treated with tricyclic antidepressants or antiepileptics [1]. Unfortunately, the use of these oral drugs may be limited due to accompanying adverse events, such as drowsiness, constipation, and/or urinary retention. Caution is advised with the use of these drugs, because constipation and urinary retention are common problems in MS patients [2, 3]. Topical antidepressants might reduce these dose-limiting adverse events, while still offering adequate analgesia [4, 5].

We describe here a double blind placebo controlled n-of-1 study in a patient with MS suffering from severe central neuropathic pain, which was effectively treated with topical amitriptyline cream without any lasting dose-limiting adverse events.

## 2. Case Report

A 62-year old woman with 14 years of primary progressive MS has been wheelchair-bound for eight years and suffered from severe neuropathic pain in the upper arms and in the left foot. The pain was characterized by pricking, tingling, numbness, and sometimes electric shocks. The average pain score, 8 on the 11-point numerical rating scale (NRS), was reduced to 6 after the usage of oral pregabalin 450 mg daily. On previously prescribed oral amitriptyline 40 mg daily she experienced intolerable adverse events, such as continuous drowsiness and tiredness. Her other complaints, such as urine retention, constipation, and spasms were successfully treated with solifenacin, polyethylene glycol, and baclofen, respectively. In May 2010, while on pregabalin treatment, the patient developed a severe burning pain in her left foot, scoring 9 on NRS. The patient also experienced electric shocks and could not bear the contact with bed sheets (allodynia).

We prescribed topical amitriptyline 5% cream to apply 3 mL once daily to the painful foot. Thirty minutes after application the patient did not experience allodynia anymore. However, when the initial pain score was higher than 5 on NRS, the burning pain was only partially reduced. Therefore, we prescribed amitriptyline 10% cream, which relieved the burning pain completely (from 9 to 0 on the NRS) already 10 minutes after each application. The relief lasted the entire day. The patient reported tiredness, as the only adverse event of the topical amitriptyline 10% cream, but even this symptom disappeared in two weeks.

In order to determine whether pain alleviation was due to topical or to systemic effect, we suggested distant application. After application to the nonpainful foot, analgesia could also be observed in the painful foot, although 15 minutes later. Due to her physical impairments the patient was not able to apply the topical amitriptyline to her painful foot. Therefore, we advised her to apply topical amitriptyline cream to her inner forearms, which turned out to have the same effect as direct application to the nonpainful foot. Also the pricking and tingling neuropathic pain in her upper arms and her left leg responded to the topical amitriptyline 5% cream, though only partially. Then however, direct application of amitriptyline 5% cream, once a day on her upper arms and her left foots reduced this pricking and tingling pain from 8 to 0 on NRS during the second day.

To further inquire into the magnitude of the placebo response, we designed a double blind placebo controlled crossover n-of-1 study, comparing topical placebo cream to amitriptyline 5%. The compounding pharmacist blinded the two creams in two separate tubes, and named the creams A and B, respectively. Successful treatment was defined based on the following criteria:

(1) relevant clinical pain reduction measured on NRS,
(2) successfully unblinding the treatment,
(3) no or minimal need to use escape medication.

The instruction was to apply once daily 3 mL cream to the arm from one tube during one week, with an escape possibility of amitriptyline 10%, and to alternate the tubes every week following the pattern ABAB. The result was that the pain disappeared after application of cream B, while most of the time cream A did not reduce the pain. The patient could correctly unblind both creams, determining B as active. She noted that in the week of using the active cream no allodynia was present, with a carryover effect of one day. She did not need to use the escape medication in the week of using the active cream, though she used frequently escape medication in the placebo weeks, especially when the pain scores were above 4 on NRS. As a matter of fact, the patient could correctly identify the active cream as 5%, based on her previous experiences with both 5% and 10% creams. Furthermore, the patient did not experience any adverse events.

## 3. Discussion

To our knowledge this is the first report of treating central neuropathic pain due to MS with topical amitriptyline cream. This double blind placebo controlled n-of-1 experiment demonstrates that most of the pain reduction could be attributed to the active compound. The use of amitriptyline 5% and amitriptyline 10% cream as escape medication was chosen, because of the profound pain reducing effect in former observations [6, 7]. The fact that the burning pain disappeared after distant application suggests a systemic effect. Despite that pregabalin 450 mg daily reduced the pricking and tingling pain, severe burning pain with allodynia developed. In contrast, a complete reduction of this burning neuropathic pain with allodynia was achieved after adding topical amitriptyline. This observation might suggest that distinct characteristics of the central neuropathic pain respond differently to different types of analgesics, supported by sparse research [8, 9]. Systematic clinical examination and research will clarify this issue in the future.

Also a local analgesic effect might play a role, because the already existing neuropathic pain in arms and left leg (mainly pricking and tingling) vanished completely only after local application. Due to the refractory and chronic character of this pain, placebo effect is less likely to contribute considerably in the positive effect. The reduction of ectopic pulses from nociceptors could be the explanation of the complete disappearance after topical amitriptyline [10]. Degeneration of some spinothalamic tract neurons can trigger pathological activity in neighboring intact afferents through the release of inflammatory mediators and neurotrophic factors. In addition, also the loss of intraspinal and descending inhibitory pathways elicits neuronal hyperexcitability [11]. Moreover, the degeneration of myelinated efferent fibers could induce spontaneous activity in uninjured C-fiber afferents [12]. To clarify which part of the analgesic response can be attributed to either systemic or local effect, in future studies serum levels of amitriptyline should be measured.

Due to controlled release characteristics of the amitriptyline cream, persistent peak-dose adverse events were absent.

Only a few case reports are available on topical analgesics in central neuropathic pain, describing the use of lidocaine 5% patches; menthol cream, and the combination cream of capsaicin 0.075%, lidocaine 3%, and isosorbide dinitrate 0.4% [13–16]. In peripheral neuropathic pain topical amitriptyline, sometimes combined with ketamine, is evaluated with mixed results, probably due to the insufficient concentration and/or a suboptimal vehicle [17, 18].

 Our paper suggests a systemic as well as a local analgesic effect of topical amitriptyline cream. More research is urgently needed for this interesting therapeutic option in the treatment of central neuropathic pain.
